# An application of Random Forests to a genome-wide association dataset: Methodological considerations & new findings

**DOI:** 10.1186/1471-2156-11-49

**Published:** 2010-06-14

**Authors:** Benjamin A Goldstein, Alan E Hubbard, Adele Cutler, Lisa F Barcellos

**Affiliations:** 1 Division of Biostatistics, School of Public Health, University of California, Berkeley, CA, USA; 2 Department of Mathematics & Statistics, Utah State University, Logan UT, USA; 3 Genetic Epidemiology and Genomics Laboratory, Division of Epidemiology, School of Public Health, University of California, Berkeley, CA, USA

## Abstract

**Background:**

As computational power improves, the application of more advanced machine learning techniques to the analysis of large genome-wide association (GWA) datasets becomes possible. While most traditional statistical methods can only elucidate main effects of genetic variants on risk for disease, certain machine learning approaches are particularly suited to discover higher order and non-linear effects. One such approach is the Random Forests (RF) algorithm. The use of RF for SNP discovery related to human disease has grown in recent years; however, most work has focused on small datasets or simulation studies which are limited.

**Results:**

Using a multiple sclerosis (MS) case-control dataset comprised of 300 K SNP genotypes across the genome, we outline an approach and some considerations for optimally tuning the RF algorithm based on the empirical dataset. Importantly, results show that typical default parameter values are not appropriate for large GWA datasets. Furthermore, gains can be made by sub-sampling the data, pruning based on linkage disequilibrium (LD), and removing strong effects from RF analyses. The new RF results are compared to findings from the original MS GWA study and demonstrate overlap. In addition, four new interesting candidate MS genes are identified, *MPHOSPH9, CTNNA3, PHACTR2 *and *IL7*, by RF analysis and warrant further follow-up in independent studies.

**Conclusions:**

This study presents one of the first illustrations of successfully analyzing GWA data with a machine learning algorithm. It is shown that RF is computationally feasible for GWA data and the results obtained make biologic sense based on previous studies. More importantly, new genes were identified as potentially being associated with MS, suggesting new avenues of investigation for this complex disease.

## Background

Genome-wide association (GWA) studies are a well-established approach for identifying genetic regions of interest for many common complex diseases and traits [1]. These studies are characterized by examining genetic information from thousands of individuals, at hundreds of thousands of loci across the human genome known as single nucleotide polymorphisms (SNPs). The standard assumption is that either variation at particular loci leads to changes in biological function, which in turn leads to disease, or that associated loci are in linkage disequilibrium (LD) with other disease causing variants. By examining genotypes derived from individuals with and without the disease or trait of interest, one can discern such variation. This is typically done by performing a marginal chi-square test with some control for multiple testing. However, since each causal SNP will confer risk under an unknown and different genetic model (i.e. additive, dominant, recessive), and may also interact with other SNPs (epistasis), a marginal test will be a less successful approach for finding the association [2]. Ideally, one would simply test all possible genetic models of association, including those for interaction. However, in the context of a GWA study, this is not computationally feasible.

Recent emphasis has been on the use of machine learning techniques to identify potential causal variants. Such techniques include logic regression [3], multi-dimensional reduction (MDR) [4], support vector machines (SVM) [5], and Random Forests (RF) [6]. While these techniques are each unique, they have a shared characteristic whereby each algorithm searches over a transformed version of the feature space attempting to find the optimal solution to the problem while minimizing some empirical risk. Importantly, the algorithms make minimal assumptions about the causal mechanism. This means these algorithms may be more suited for identifying variants where the causal mechanism is unknown and complex, as is the case with complex genetic diseases.

Each of these methods has utility for finding structure in genetic data, where the best algorithm will depend on the true nature of the underlying association. However, the focus of the current study is RF because of the ability of this method to identify variables of interest from very large datasets. Equally important, RF is a relatively straightforward algorithm, both to understand and interpret. Unsurprisingly, there has been a slow but steady use of RF in the genomic literature since its introduction in 2001 [6-12].

**Figure 1:**
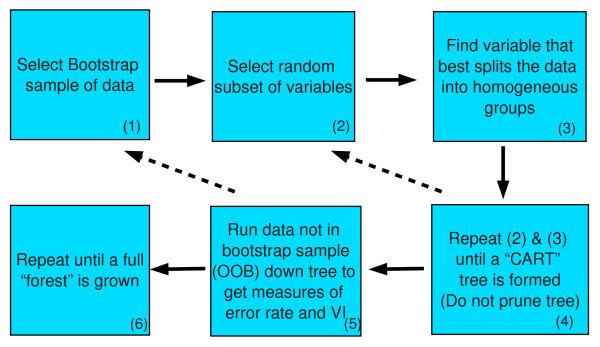
**Random Forests Algorithm**. The RF algorithm begins by selecting a bootstrap sample of the data (1). A random subset of the variables is selected (2) and searched over to find the optimal split (3). This is repeated until an unpruned CART tree is formed (4). The data not part of the bootstrap sample is run down the tree to derive the error rate and measures of VI (5). This is repeated until a full forest is grown (6).

RF was first introduced by Leo Breiman [13] and is a natural extension of his previous work on classification and regression trees (CART) [14] and bootstrap aggregating (or bagging) [15]. CART is an effective tool for building a classifier, but tends to be data dependent, where even small data changes can result in different tree structures. Bagging is a process whereby data are sampled with replacement and the classifier is grown using this bootstrap sample. After many iterations, results are aggregated over all trees to create a less variable classifier with a lower prediction error when compared to the original classifier. In bagging, the variance reduction is limited by the correlation between trees; as correlation is decreased or minimized, the potential for reduction is increased. The RF algorithm (see Figure 1) begins by bagging CART trees. To reduce the correlation between trees, instead of searching over all p variables at each node for the optimal split, a search is performed over a random subset, m ≤ p, at each node. The algorithm continues to split the data until no further splits are possible, either because the node is pure (all of one class), or there are no more variables upon which to split. While the CART algorithm calls for the tree to be pruned for increased stability, RF leaves the tree unpruned, as bagging is used to decrease the variance created by the lack of pruning.

An aspect of the bagging procedure is that a natural, internal error rate is created. Within each bootstrap sample, approximately 37% of the original data will be unselected, referred to as the out-of-bag (OOB) sample [16]. RF passes OOB samples down the tree to obtain a class prediction. After the full forest is grown, the class predictions are compared to the true classes generating the out-of-bag error rate (OOB-ER). This error-rate can be used to compare the prediction accuracy of one set of inputs to another, behaving similarly to cross-validation [17].

An appeal of RF is that the forest of trees contains a large amount of information about the relationship between the variables and observations. This information can be used for prediction, clustering, imputing missing data, and detecting outliers. Of great interest to genetic epidemiologists, is the ability of RF to identify 'important' variables. After each OOB sample is passed down the tree to produce a prediction error for the sample, one then permutes each variable in the tree across samples, and passes the same observation down the tree again. Any increase in misclassification helps determine the importance of that variable. This type of variable importance (VI) can be derived from disparate variable types (categorical, ordinal, continuous), and makes no assumptions about the data generating distribution for the outcome. However, unlike a formal hypothesis, it is best to consider the output of a RF analysis as a rank ordering of important variables worthy of further investigation, not as a list of variables with a known Type I error rate.

Utilization of RF requires choosing between three tuning parameters: (1) number of trees to grow *(ntree)*, (2) number of variables to select per-node *(mtry)*, and in the case of classification, (3) class weights. While most applications in the literature have successfully implemented RF using default settings, applying RF to large GWA datasets is more complicated. Few studies have examined the various tuning parameters. The two most comprehensive reviews concluded that RF predictions were stable and robust to small fluctuations in tuning parameters settings, but often there were optimal settings [7,18]. While both studies provide useful information, the largest dataset examined by each contained only 9,868 predictors and 78 observations. This is obviously much smaller than the data analyzed in a typical GWA study.

Further complicating RF analysis, beyond the large feature space, is that GWA data tend to be highly correlated, with potentially, many regions of LD among SNPs. Also, the data are assumed to be highly sparse, meaning there is an apriori assumption that the vast majority of SNPs will not be associated with the disease. While many of these issues have been discussed in the literature, none have been considered in the context of a large GWA dataset. Moreover, many of the strategies one would employ with smaller data sets (e.g. permutation, cross-validation etc.) are not feasible due to computational constraints. Instead of working with simulated data which can be less realistic, we investigated the application of RF using a large multiple sclerosis (MS) GWA study dataset comprised of cases and controls. The aim of the current study was two-fold: (1) to illustrate how one would go about tuning RF for a particular GWA analysis, and (2) to determine whether RF would duplicate results found in the original MS GWA study, as well as identify any new loci of interest.

## Methods

### Genotypes

Data were derived from a 2007 MS case-control study conducted by the International Multiple Sclerosis Genetics Consortium [19] and were comprised of genotypes for a total of 325,807 SNPs (Affymetrix GeneChip Human Mapping 500K array) in 931 MS cases and 2,431 controls (n = 3,362). Stringent quality control (QC) analyses were applied to the dataset as previously described, including the removal of population outliers [19]. SNPs with greater than 10% missing data were removed. The genetic inflation factor was 1.06, indicating negligible population stratification [19].

Less than 1% of the genetic data contained missing values. There are a few different ways missing data can be handled within RF. However, since the data were derived from a dense SNP marker panel and had minimal missingness, any missing values were imputed with Beagle 2.13 [20]. Allelic data were then recoded into genotype format using PLINK 1.05 [21], producing three categories for each SNP (0, 1 and 2 copies of the minor allele). Since the optimal binary split is found at each node, this allows for the algorithm to be agnostic to recessive, dominant or additive effects. An allelic chi-square test (df = 1) was performed to calculate marginal associations for comparison.

### RF Implementation

The RF code was originally written in Fortran by Breiman and Cutler. There is also an R package randomForest based on the same Fortran code [22]. Neither implementation could be used for the large GWA dataset in the current study. The original RF code has been licensed to Salford Systems [23], and they recently optimized the Fortran version, v.6.4.0.179, for application to large datasets. In preliminary testing of small datasets, similar results were found between the three implementations of RF (data not shown). RF was implemented in a server environment with 8 2/GHz cpus and 32 GB of memory. Run time was dependent on data size and *mtry*, ranging from a few seconds per tree to over 10 minutes per tree (~ 1 week for a full forest).

### Tuning Parameters Considered

#### Number of variables to choose per node (mtry)

The primary tuning parameter in RF is the number of variables to search at each node *(mtry)*. This parameter controls the bias-variance trade-off. Searching over fewer variables per node will produce less correlated trees, reducing the overall variance of the prediction. However, this will also decrease the accuracy of each individual tree, increasing the bias. The *mtry *can also be viewed as controlling the complexity of the model, with a smaller value leading to a more complex, less sparse solution (see below). Breiman originally suggested choosing the int(log _2 _*p *+ 1) of the number of predictors per node. In the R implementation, the default value is the square root of the number of predictors.

For a GWA dataset, this would entail examining approximately 550 SNPs per node. As noted by Breiman, when there are many weak predictors, this number may need to be increased. It has also been noted that *mtry *is more important for VI calculation than for prediction, and that with sparse data, *mtry *= p leads to greatest stability [18]. A coarse search for the optimal *mtry *was performed in the current study using *mtry *values of 1, , 0.1p, 0.5p and p. The parameterization that produced the lowest final OOB-ER was chosen as the optimal *mtry.*

#### Number of Trees to Grow (ntree)

Another important consideration is how many trees to grow. This is also a dataset dependent factor, where stronger predictors lead to quicker convergence. While for prediction purposes few trees are often necessary, and the OOB-ER will generally converge rapidly, for VI, more trees will generally lead to refinement and stability in VI [18].

The main trade-off with growing a larger number of trees is the computation cost required. In the current study, trees were grown until the OOB-ER stabilized. Additional trees were then grown to ensure stability.

#### Weighting

The final tuning parameter, which was not considered in this analysis, is weighting. In classification, with uneven classes, an unweighted classification scheme will be biased towards the majority class. The typical strategy is to re-weight the classes so that they are balanced, the practice used within the Salford Systems implementation of RF, and the default in the R implementation. Unfortunately, class weighting cannot be altered in the Salford Systems version, so it could not be tested as a tuning parameter. However, internal testing on a more flexible version of RF showed no added benefit to changing the weighting.

### Data Configurations

#### Sparsity Pruning

As noted, it is expected that the vast majority of SNPs in a GWA study do not impact risk for disease, and therefore, are simply noise. The goal of any algorithm should be to separate noise from signal, providing a sparse solution. A sparse solution is indicated when the VI is either 0 or negative. Such a VI indicates that the variable was either never selected into a tree, or when it was selected, permutation did not increase the prediction error. Sparse solutions provide a convenient way to remove unimportant data from the analysis.

Sparsity is a function of both *mtry *and *ntree*, with a higher *mtry *leading to greater sparsity and a higher *ntree *leading to less sparsity. One proposed strategy is to sequentially remove genes by dropping the bottom 20% or 50%, and perform successive runs until there is a noticeable increase in prediction error [7]. Utilizing the natural sparseness in the dataset, the results of each RF run were examined and sparse SNPs were dropped. The RF analysis was then re-run until prediction error stabilized. While this will give a biased estimate of the prediction error for the model [24], it can still be used to judge model quality. This sub-sampling process was repeated in the current study until the final OOB error-rate stabilized or increased.

#### Removing Strong Associations

RF searches over multiple variables finding solutions based on joint and conditional effects. Variable(s) with strong effects may mask weaker, yet important effects. It is well established that the HLA region within the major histocompatibility complex (MHC) on chromosome 6p is strongly associated with MS [25]. Therefore, to search for weaker non-MHC effects, RF analysis was performed in the current study after removing chromosome 6p marker data.

#### Linkage Disequilibrium

An important consideration when applying RF to GWA data is the large degree of LD among SNPs. VI is calculated from the number of trees in which a variable appears. Therefore, two SNPs that are in perfect LD will appear in trees about half as often as each individual one may appear by itself, effectively lowering the VI of each SNP. While this does not present a problem for prediction, it can skew the VI rankings [18]. Two proposed solutions have been to calculate VI independently of the number of trees in which the variable appears [10] or as conditional on other variables in the tree [26].

PLINK [21] provides two methods of LD pruning based on *r *^2 ^and *R *^2^. *r *^2 ^is a traditional pairwise LD measure, though not based on phased haplotypes. *R *^2 ^is the multiple correlation coefficient based on a sliding window. Using PLINK, SNPs with a multiple correlation coefficient (*R *^2^) of 0.99, 0.90, 0.80, 0.50 and 0.33 were removed from the MS case-control dataset for comparison. This resulted in pruning between 22% and 76% of the original data which had the side benefit of increasing computational efficiency.

### Reliability of Results Obtained from RF

Since RF is a Monte-Carlo process, random variation may influence VI results, particularly if enough trees are not grown. While, work has indicated that RF results are relatively stable [18] and our own internal testing has confirmed this, it is important to grow large forests and do multiple runs when possible. Reliability of final RF results was examined by re-running RF with the final dataset configuration, parameterization and sub-sampling process, changing just the seed in the random number generator. While more than one re-run would be ideal, the VI measures are unlikely to be unstable given that two runs were performed.

### Comparison of RF Results to Original GWA study

The original MS GWA study identified, with replication, 16 SNPs across 13 genes as associated with MS [19]. An important consideration for the current study was whether RF could identify additional genes of interest, as well as duplicate the original findings based on univariate testing. Duplication was considered present when a SNP identified by RF was: (1) among the original 16 SNPs, or (2) a SNP that was tagged by one of the 16 SNPs identified in the original GWA study. PLINK was used to identify tagged SNPs using an *r *^2 ^threshold of 0.5.

### Analysis Strategy

**Figure 2:**
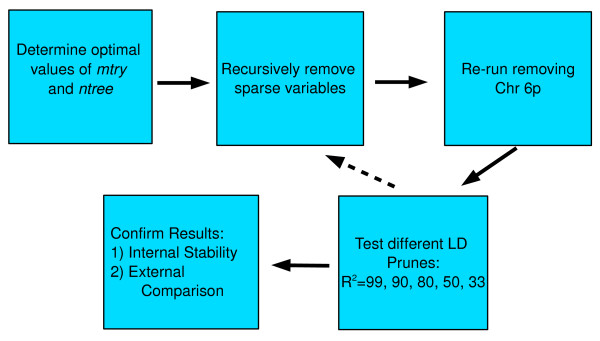
**Analysis Flow**. Flow Plan for RF analysis. The full MS case-control dataset was analyzed, searching for the optimal *mtry *&*ntree*, along with sparsity pruning, as necessary. Two runs were then conducted, one without any 6p genotypes, and one with data for a single 6p SNP. Finally, LD pruning was explored. After the best data configuration was found, RF analysis was re-run to examine stability of results. The final RF results were compared to the original GWA results [19].

**Figure 3:**
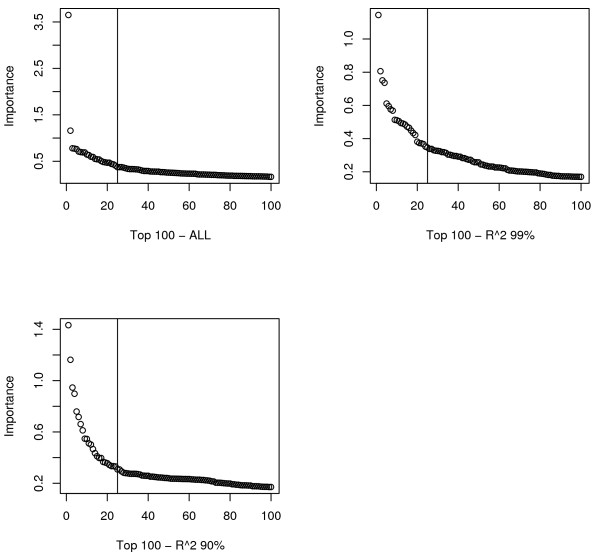
**Scree Plots for top 100 RF VI measures**. The three plots represent the VI measures for the full dataset with chromosome 6p data removed, the *R *^2 ^= 0.99 run and the *R *^2 ^= 0.90 run. An "elbow" is present in all three plots around 25 markers (designated with the vertical line).

Figure 2 presents the analysis plan. The primary method for choosing tuning parameters was minimization of the OOB-ER, as this is the best indication of model quality. Determining how many results to report is more subjective since the VI measure does not constitute a formal hypothesis test. To help guide interpretation of RF results for follow-up, we plotted VI scores. A sloping line with an "elbow" (Figure 3) was observed most often around the top 25, so this was chosen as the cutoff for an important result.

## Results

### Tuning Parameters

#### Number of variables to choose per node (mtry)

**Figure 4:**
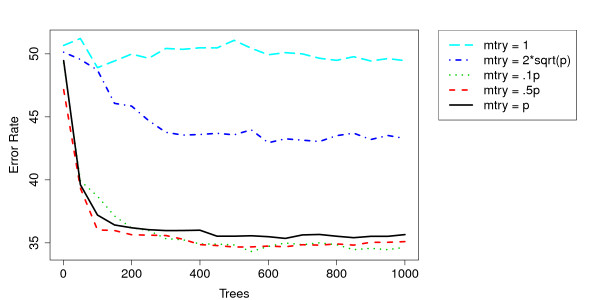
**Convergence of Error Rate Across Different mtrys**. An examination of the error-rate across different *mtrys*. The larger *mtrys *of .1p and above clearly lead to a much lower error rate than the more traditional lower values. .1p seems to minimize the overall OOB error-rate though not by much. Convergence seems to occur around 200 - 400 trees.

The first parameter considered was *mtry *since this has the greatest impact on the OOB-ER. Figure 4 shows the OOB-ER for different values of *mtry*. The typically suggested value of *mtry *of around  is not sufficient for GWA data, as the OOB-ER is minimized with an *mtry *around .1p. Among the higher *mtry *values, there was little distinction between them with regard to OOB-ER.

**Figure 5:**
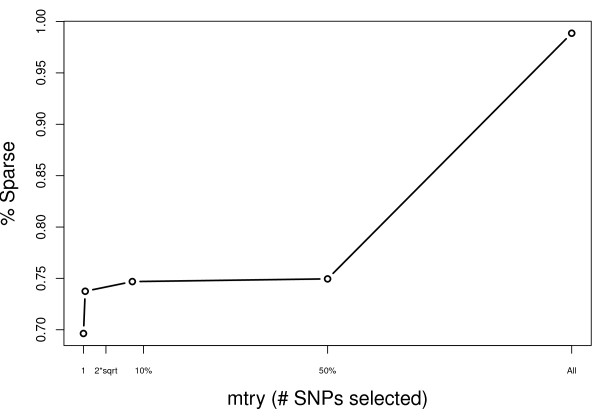
**Sparsity of SNPs across mtry**. As expected, sparsity increases as a function of *mtry*. There is the most dramatic increase after moving from an *mtry *of .5p to p.

Another consideration is the sparsity induced by the *mtry *factor. As expected, sparsity increases with *mtry*, though this is most dramatic after increasing to *mtry *= p (Figure 5).

#### Number of Trees to Grow (ntree)

Using *mtry *= .1p, forests of size 50, 250, 500, 1,000, 1,500 and 2,000 trees were grown. It is clear that the OOB-ER leveled off around 250 trees (see Figure 4) and 1,000 trees was used as a reliable forest size. However, for datasets without chromosome 6p and only weak predictors (see below), it took more than 4,000 and sometimes 8,000 trees for convergence. In those cases, 5,000 and 10,000 trees, respectively, were grown. More trees led to a less sparse result, as expected, with nearly a linear decrease through 2,000 trees.

### Data Configurations

#### Sparsity Pruning

When using the full dataset for RF analysis, SNPs within the HLA region of chromosome 6p were consistently selected as the most important variables (Table 1). This is not surprising, as some SNPs in that region had a marginal Χ^2^-statistic as large as 274. The final error rate of 35% is identical to a simple classification based just on genotypes for the three most highly associated SNPs (rs3129900, rs3129934, rs9370986). RF results based on analysis of all SNPs from chromosome 6p resulted in the same 35% error rate.

**Table 1:** Top SNPs identified by Random Forests in MS case-control dataset

Chr	SNP	Gene	MAF	RF Rank	CHISQ	P-Value
6	rs3129900	*C6orf10*	0.17	1	272.2	3.75 * 10^-61^

6	rs3129934	*C6orf10*	0.17	2	274.4	1.28 * 10^-61^

6	rs9270986	*HLA Tag SNP*	0.17	3	274.6	1.14 * 10^-61^

6	rs3129768	HLA-DQA* (70 bp)	0.20	4	238.9	3.14 * 10^-53^

6	rs2647046	HLA-DQA2* (8.5 kb)	0.39	5	113.9	1.38 * 10^-26^

6	rs3129932	*C6orf10*	0.23	6	219.8	1.02 * 10^-49^

6	rs9275572	HLA-DQA2* (2.1 kb)	0.42	7	101.5	7.24 * 10^-24^

6	rs3131294	*NOTCH4*	0.14	8	215.4	9.26 * 10^-49^

6	rs910049	*C6orf10*	0.24	9	222.2	2.98 * 10^-50^

6	rs2894249	*C6orf10*	0.23	10	220.7	6.28 * 10^-50^

6	rs3135377	HLA-DRA* (80.6 kb)	0.21	11	217.9	2.60 * 10^-49^

6	rs9469220	HLA-DQA2* (18.5 kb)	0.50	12	99.2	2.28 * 10^-23^

6	rs7194	*HLA-DRA*	0.40	13	129.7	4.69 * 10^-30^

6	rs6457620	HLA-DQB1* (137.5 kb)	0.49	14	96.03	1.13 * 10^-22^

6	rs3130287	*TNXB*	0.15	15	181.2	2.72 * 10^-41^

6	rs6457617	*HLA-DQB1 (137.4 kb)*	0.49	16	96.03	1.13 * 10^-22^

6	rs6936204	C6orf10* (14.6 kb)	0.36	17	113.3	1.83 * 10^-26^

12	rs1805755	*M6PR*	¡ .01	18	73.42	1.05 * 10^-17^

12	rs1716167	*MPHOSPH9*	0.21	19	22.38	2.23 * 10^-6^

7	rs17708673	*C7orf25 (106.2 kb)*	0.16	20	6.357	1.17 * 10^-2^

6	rs9268877	HLA-DRA* (126.3 kb)	0.42	21	74.57	5.85 * 10^-18^

6	rs9276440	*HLA-DQA2*	0.45	22	83.75	5.63 * 10^-20^

6	rs2621383	HLA-DOB* (825.5 kb)	0.37	23	82.72	9.44 * 10^-20^

22	rs80515	FAM19A5* (1.4 mb)	0.10	24	3.751	5.28 * 10^-2^

20	rs2425754	CDH22* (580.3 kb)	0.15	25	4.193	4.06 * 10^-2^

The top 25 SNPs from RF analysis of the whole dataset are shown above. Most of the top SNPs are on chromosome 6p within the HLA region. The minor allele frequency (MAF) is derived from controls and the *χ*^2^-statistic is from univariate testing. *Indicates that the gene is the closest gene with distance.

#### Removing Chromosome 6p

After removing all SNPs on chromosome 6p (p = 8,335), the initial run of 317,472 SNPs produced an error-rate of 48% after 1,000 trees, and using both an *mtry *of .1p and p. The resulting forest based on *mtry *of .1p was 74% sparse (82,527 SNPs retained). Using *mtry *= p, the forest was 99% sparse (4,219 SNPs retained).

For the *mtry *= p run, re-running RF analysis with the reduced dataset produced an error-rate of 26%, and required approximately 4,000 trees to converge. Repeating this sub-sampling process two more times produced an error-rate of 21%. After a fourth run, the OOB error-rate remained at 21%, suggesting that three sub-samples were sufficient. For the 10% run, the final OOB error-rate was 37% and contained 25,000 SNPs.

Overall, results suggest there is predictive structure (differences between MS cases and controls) beyond chromosome 6p, and that aggressive pruning of the initial *mtry *= p is more effective for discovering that structure. The top 25 SNPs derived from RF analysis without chromosome 6p markers are shown in Table 2.

**Table 2:** Top SNPs identified by Random Forests in MS case-control dataset without 6p data

Chr	SNP	Gene	MAF	RF Rank	CHISQ	P-Value
12	rs1805755	*M6PR*	< 0.01	1	73.42	1.05 * 10^-17^

7	rs6467970	SEMA3A* (44.1 kb)	0.19	2	19.71	9.00 * 10^-6^

10	rs10823051	*CTNNA3*	0.16	3	17.61	2.71 * 10^-5^

1	rs10754012	RGS1* (3.3 mb)	0.23	4	22.24	2.41 * 10^-5^

12	rs1716167	*MPHOSPH9*	0.21	5	22.38	2.23 * 10^-6^

6	rs1015340	*PHACTR2*	0.47	6	14.86	1.16 * 10^-4^

10	rs7068990	PPAPDC1A* (137.6 kb)	0.23	7	17.65	2.65 * 10^-5^

8	rs1466526	FAM164A* (86.0 kb)	0.25	8	15.60	7.84 * 10^-5^

6	rs1040638	*PHACTR2*	0.48	9	13.82	2.01 * 10^-4^

7	rs16217	NPY* (292.9 kb)	0.26	10	7.14	7.53 * 10^-3^

12	rs1106240	*PITPNM2*	0.20	11	18.71	1.52 * 10^-5^

8	rs4739135	FAM164A* (98.8 kb)	0.19	12	16.33	5.34 * 10^-5^

18	rs4798684	ADCYAP1* (19.8 kb)	0.30	13	13.41	2.51 * 10^-4^

6	rs1015341	*PHACTR2*	0.47	14	14.88	1.14 * 10^-4^

12	rs2695478	*MPHOSPH9*	0.20	15	17.30	3.19 * 10^-5^

1	rs11800848	*EVI5*	0.26	16	19.04	1.28 * 10^-5^

9	rs6993386	*IL7*	0.32	17	17.95	2.27 * 10^-5^

6	rs6915752	*PHACTR2*	0.45	18	17.71	2.57 * 10^-5^

20	rs2223712	BTBD3* (3.6 kb)	0.19	19	11.86	5.73 * 10^-4^

10	rs7092549	PPAPDC1A* (140.0 kb)	0.23	20	17.06	3.62 * 10^-5^

6	rs9376783	*PHACTR2*	0.45	21	17.26	3.27 * 10^-5^

17	rs17652139	CCL2* (3.0 mb)	0.23	22	11.81	5.88 * 10^-4^

7	rs740295	*MGC87402*	0.31	23	7.89	4.96 * 10^-3^

5	rs156823	*ARL15*	0.47	24	12.13	4.97 * 10^-4^

18	rs7241142	ADCYAP1* (20.7 kb)	0.30	25	10.81	1.01 * 10^-3^

The top 25 SNPs from RF analysis of the dataset without chromosome 6 SNPs are shown above. The minor allele frequency (MAF) is derived from controls and the *χ*^2^-statistic is from univariate testing. *Indicates that the gene is the closest gene with distance.

#### Linkage Disequilibrium

**Figure 6:**
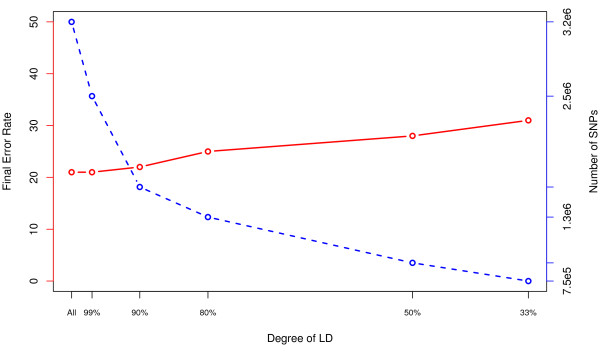
**Error Rate Across LD Prunes**. In the red line we see the OOB error rate across the different LD prunes. There is little information lost going from the full data to pruning at 99% and even 90%. Thereafter there is more loss of information. The blue line shows the number of SNPs that were in each RF analysis.

The final consideration was the effect of pruning SNPs based on LD. The dataset without any markers for chromosome 6p was used and the same sub-sampling strategy was followed. Figure 6 shows final error-rates for the six LD configurations investigated, along with the full dataset. The number of SNPs in each configuration is included.

While pruning past an *R*^2 ^of 0.90 (LD90) results in a higher final error-rate and suggests a loss of information, it is hard to determine which approach is best when solutions based on full data, LD99 and LD90 are compared. Examination of the top 25 SNPs from the three configurations (full, LD99 and LD90; Tables 2, 3 and 4), reveals that most of the SNPs were located within a gene (14, 14, and 15 respectively). However, the LD90 solution identified SNPs within more unique genes (14) compared to the other configurations (9 and 11). In addition to identification of potentially functional SNPs, the majority of top results show strong marginal associations (p ≃ 10^-5^) but do not meet established criteria for genome-wide significance [27]. When the top 25 SNP results from each configuration were compared, both overlapping and unique genes are observed. Genes not previously associated with MS were among the top hits, specifically *CTNNA3, MPHOSPH9, PHACTR2*, and IL7.

**Table 3:** Top RF SNPs in the MS case-control dataset with LD pruning R^2 ^= 0:99

Chr	SNP	Gene	MAF	RF Rank	CHISQ	P-Value
7	rs6467970	SEMA3A* (44.1 kb)	0.19	1	19.71	9.00 * 10^-6^

8	rs1466526	FAM164A* (86.0 kb)	0.25	2	15.60	7.84 * 10^-5^

12	rs1716167	*MPHOSPH9*	0.21	3	22.38	2.23 * 10^-6^

10	rs10823051	*CTNNA3*	0.16	4	17.61	2.71 * 10^-5^

1	rs11800848	*EVI5*	0.26	5	19.04	1.28 * 10^-5^

17	rs17652139	CCL2* (3.0 mb)	0.23	6	11.81	5.88 * 10^-4^

6	rs1015341	*PHACTR2*	0.47	7	14.88	1.14 * 10^-4^

7	rs16217	NPY* (292.9 kb)	0.26	8	7.14	7.53 * 10^-3^

1	rs12743520	*EVI5*	0.26	9	18.61	1.61 * 10^-5^

6	rs1040638	*PHACTR2*	0.48	10	13.82	2.01 * 10^-4^

18	rs4798684	ADYCAP1* (19.8 kb)	0.30	11	13.41	2.51 * 10^-4^

8	rs6993386	*IL7*	0.32	12	17.95	2.27 * 10^-5^

1	rs2760524	RGS1* (3.3 mb)	0.19	13	20.00	7.76 * 10^-6^

10	rs7092549	PPAPDC1A* (140.0 kb)	0.23	14	17.06	3.62 * 10^-5^

20	rs2223712	BTBD3* (3.6 kb)	0.19	15	11.86	5.72 * 10^-5^

7	rs740295	*MGC87402*	0.31	16	7.90	4.96 * 10^-5^

1	rs282177	*RPS6KA1*	0.26	17	17.01	3.72 * 10^-5^

6	rs6570578	*PHACTR2*	0.45	18	17.00	3.72 * 10^-5^

10	rs7068990	PPAPDC1A* (137.6 kb)	0.23	19	17.65	2.65 * 10^-5^

1	rs1359062	RGS1* (3.3 mb)	0.19	20	18.49	1.71 * 10^-5^

2	rs698853	*LOC100302652*	0.28	21	16.75	4.26 * 10^-5^

7	rs156293	NPY* (313.8 kb)	0.22	22	9.56	1.99 * 10^-5^

16	rs6499946	*KLKBL4*	0.22	23	12.83	3.41 * 10^-4^

2	rs7583622	*ASB3*	0.23	24	17.07	3.60 * 10^-5^

20	rs17408919	*PAK7*	0.23	25	11.63	6.50 * 10^-4^

The top 25 SNPs from RF analysis of the dataset without chromosome 6 SNPs are shown above in the *R*^2 ^= 0:99 runs after 3 sub-samplings. Results are similar to analysis of full dataset. *Indicates that the gene is the closest gene with distance.

**Table 4:** Top RF SNPs in the MS case-control dataset with LD pruning *R*^2 ^= 0.90

Chr	SNP	Gene	MAF	RF Rank	CHISQ	P-Value
7	rs6467970	SEMA3A* (44.1 kb)	0.19	1	19.71	9.00 * 10^-6^

8	rs1466526	FAM164A* (86.0 kb)	0.25	2	15.60	7.84 * 10^-5^

10	rs10823051	*CTNNA3*	0.16	3	17.61	2.71 * 10^-5^

1	rs10754012	RGS1* (3.3 mb)	0.23	4	22.24	2.41 * 10^-6^

6	rs1040638	*PHACTR2*	0.48	5	13.82	2.01 * 10^-4^

8	rs4739135	FAM164A* (98.8 kb)	0.19	6	16.33	5.34 * 10^-5^

18	rs4798684	ADCYAP1* (19.8 kb)	0.30	7	13.41	2.51 * 10^-4^

10	rs7092549	PPAPDC1A* (140.0 kb)	0.23	8	17.06	3.62 * 10^-5^

14	rs10483442	*NPAS3*	0.19	9	13.88	1.95 * 10^-4^

20	rs2223712	BTBD3* (3.6 kb)	0.19	10	11.86	2.71 * 10^-4^

12	rs1106240	*PITPNM2*	0.20	11	18.71	1.52 * 10^-5^

5	rs156823	*ARL15*	0.47	12	12.13	4.97 * 10^-4^

9	rs10975130	*KANK1*	0.16	13	24.04	7.81 * 10^-6^

12	rs12578774	AACS* (1.3 mb)	0.31	14	19.67	9.19 * 10^-6^

2	rs7583622	*ASB3*	0.23	15	17.07	3.60 * 10^-5^

6	rs6570578	*PHACTR2*	0.45	16	17.00	3.73 * 10^-5^

1	rs282177	*RPS6KA1*	0.26	17	17.01	3.72 * 10^-5^

5	rs11949767	MXD3* (59.7 kb)	0.26	18	17.79	2.47 * 10^-5^

10	rs7427	*MSRB2*	0.36	19	10.64	1.10 * 10^-3^

8	rs1879818	*TRAPPC9*	0.30	20	7.35	6.72 * 10^-3^

16	rs1974876	*CCDC113*	0.15	21	10.91	9.55 * 10^-3^

17	rs11651517	*GAS7*	0.43	22	10.29	1.34 * 10^-3^

20	rs6018946	BLCAP* (581.3 kb)	0.34	23	16.85	4.04 * 10^-5^

2	rs11694785	*ARHGAP25*	0.40	24	10.15	1.44 * 10^-3^

2	rs6746541	*ATOH8*	0.35	25	16.06	6.14 * 10^-5^

The top 25 SNPs from RF analysis of the dataset without chromosome 6 SNPs are shown above in the *R*^2 ^= 0.90 runs after 3 sub-samplings. Results are similar to the analysis of full dataset, though there is more heterogenetity in the top findings, owing primarily to LD pruning. *Indicates that the gene is the closest gene with distance.

### Reliability of Results Obtained from RF

The final three data configurations (full data, LD99 and LD90) were re-analyzed, changing only the random number seed. For all three configurations, at least 19 of the top 25 SNPs were in the final results after sparsity pruning. This suggests that even after changing the seed, RF results are very stable.

### Comparison of Results to Original GWA Study

Finally, the RF results were compared with replicated results from the original MS GWA study [19] (Additional File 1). In all, 4 of 13 MS genes were directly identified by one of the three data configurations. The strongest evidence came from SNPs in *EVI5 *and *KANK1 *with a suggestion of duplication in *IL2RA*. Details including SNP rs number, minor allele frequency (MAF), and univariate analysis results are shown.

## Discussion

This study is the first application of RF, and one of few machine learning applications, to the analysis of a GWA dataset. The goals were to outline methodological considerations for applying RF to large GWA data, and to identify potential novel MS associations. Given what is currently known about the genetics of MS, it was not surprising that a strong classifier could be constructed by RF based on data for multiple SNPs within the MHC. Among the strongest effects (most important SNP predictors of MS as outcome) was rs9271366, which has been previously shown to tag DRB1*1501 with *r*^2 ^= 0.98 [28]. Interestingly, once the 6p effect was removed from analyses, a strong classifier based on non-MHC data emerged. Results suggest that sparsity pruning provides a means to discover new associations with RF, although the final error-rate is biased [24].

RF analyses consistently identified four non-MHC genes as important to distinguishing MS cases from controls. These were: *MPHOSPH9, CTNNA3, PHACTR2 *and IL7. *MPHOSPH9 *up-regulates neuronal functioning [29], and interestingly, variation within this locus has recently shown suggestive evidence for association in a much larger meta-analysis that included 2,624 MS cases and 7,220 controls [30]. *CTNNA3 *is a cell adhesion gene that has been associated with Alzheimer's disease [31]. *PHACTR2 *is involved in phosphate and actin regulation and has been implicated in Parkinson's disease [32]. Finally, *IL7 *is an important immune system gene involved in T and B cell production and has been implicated in other autoimmune diseases, notably rheumatoid arthritis, but not MS [33]. It is important to note that although SNPs within *CTNNA3, MPHOSPH9, PHACTR2 *and *IL7 *were among the top RF results, associations for these SNPs based on univariate analyses would not meet criteria for genome-wide significance [27] (Tables 2, 3, and 4). As a point of comparison, statistical power based on univariate testing was high in our dataset (n = 931 cases and 2,431 controls) for detecting an effect size per allele (or allelic odds ratio) of 1.5 (assuming MAF = 0.15-0.50 and Α = 1.5 * 10 ^-7^), However, power was quite limited to detect smaller effect sizes, for example, 1.3 or 1.2, where ∼ 5 - 30% and ∼ 0.5 - 3% power, respectively, was present. To date, replicated non-MHC MS genes have demonstrated very modest effects of 1.2 or even smaller [19,28,30]. New results from the current study will require further replication in a larger, independent dataset, but underscore the utility of using more than one analytical method to identify genetic associations.

RF results were also compared to findings from the original MS case-control study using the same dataset, with duplication defined as a either the original SNP or one tagged by that SNP among the top RF results. Two previously reported genes, *EVI5 *and *KANK1*, were among the top RF findings in the current study. There was also a suggestion of importance based on RF analyses for *IL2RA *and perhaps *CBLB.*

Methodologically, it was shown that RF can be applied to large GWA datasets, but certain standard assumptions cannot always be made. The OOB-ER was relied upon to guide decision making about tuning parameters and data configuration. Even though the focus of the current study was not prediction, this error-rate is valuable for determining the quality of RF results. First, when working with large, sparse data, the default value of *mtry *needs to be increased in order to improve learning. Even for the sub-sampled data sets, generally an *mtry *= .1p was the optimal setting. It was also found that the number of trees necessary to reach stability depended greatly on the strength of the inputs. For the data configurations with chromosome 6p genotype data, stability was reached within 250 trees, while for the data configurations without chromosome 6p data, stability was often not reached until at least 4,000 trees were generated. LD pruning can be an effective means of reducing data size without significant loss of information. Also removing sparse variables proved to be highly effective and resulted in much more efficient learning. It was established that some very strong effects (chromosome 6p) can mask weaker, yet potentially interesting effects. Prediction based on genetic data that did not include HLA region SNPs was surprisingly strong. Finally, one needs to consider the coding of the allelic data. Coding the data on a dosage scale allows for a flexible examination of genetic effects. Upon settling on a final configuration(s), doing multiple runs of RF is necessary to examine the reliability of the VI measures.

More work is needed to achieve a better understanding of the RF algorithm and how best to apply it to large GWA datasets. The theoretical basis for RF as a predictor is well understood, but less is known about VI. Unlike p-values, there is no strict criterion for distinguishing between important and non-important variables. Our decision to focus on the top 25 results was based on graphing results, and in that sense was fairly qualitative. Ideally, one would use permutation to assess the significance of the VI measures, however this is not feasible with these large datasets. Work is ongoing to determine valid cutoffs for VI measures. Also, only one form of VI was used in this analysis (permutation), but another general VI exists for classification based on the Gini criterion (the optimizing criteria used to construct the tree). Work is also ongoing to define more targeted measures of VI, particularly for SNP data. Furthermore, as discussed, LD between SNPs and other correlated data are problematic for RF due to the way VI is calculated and we are currently exploring alternative VI calculations. Finally, further work is also needed to leverage additional information from the forest of trees. Little work has been done on clustering observations in RF. The tree structure can also be used for identifying extensive regions of interactions and genetic networks and predictors important to specific disease phenotypes.

## Conclusions

This study represents one of the first successful applications of a machine learning algorithm to GWA data. Machine learning algorithms require fewer assumptions about the data generating distribution, and therefore, offer a very flexible approach to data analysis. Our results show the RF algorithm is both computationally feasible and sensible for analyses of large GWA datasets. Computation time ranged from a few minutes to a few days depending on the number of variables. Our results support findings from previous genetic studies in MS, and more importantly, new candidates emerged that strongly warrant further investigation.

A unique approach to analyzing complex genetic data is described in the current study. As other machine learning algorithms are expanded to accommodate large GWA datasets, one can apply an array of algorithms to a large dataset, and then aggregate results across methods to determine which markers or genes may be of greatest interest for future studies. Such ensemble learners are common in the machine learning literature [17], and should soon be applicable to larger genetic datasets.

## Authors' contributions

BAG, AEH, AC and LFB designed the study. BAG performed analytical work. BAG and LFB wrote the manuscript. BAG, AEH, AC and LFB contributed to discussion and edits to the manuscript. All authors have read and approve the contents of the final manuscript.

## Supplementary Material

Additional file 1 **Comparison of RF results with original GWA screen in MS cases and controls**. The top 16 SNPs identified by IMSGC [19] and corresponding rank for a tag SNPs derived from RF analysis. EVI5 and KANKI ranking the highest based on RF results, with rs10735781 having multiple tag SNPs. *The two SNPs in IL2RA serve as tag SNPs for one another. Click here for file

## References

[B1] WTCCC Genome-wide association study of 14,000 cases of seven common diseases and 3,000 shared controls Nature 2007 447 661 678 10.1038/nature05911 17554300 PMC2719288

[B2] Heidema AG Boer JM Nagelkerke N Mariman EC van der A DL Feskens EJ The challenge for genetic epidemiologists: how to analyze large numbers of SNPs in relation to complex diseases BMC Genet 2006 7 23 10.1186/1471-2156-7-23 16630340 PMC1479365

[B3] Kooperberg C Ruczinski I Identifying interacting SNPs using Monte Carlo logic regression Genet Epidemiol 2005 28 157 170 10.1002/gepi.20042 15532037

[B4] Motsinger AA Ritchie MD Multifactor dimensionality reduction: an analysis strategy for modelling and detecting gene-gene interactions in human genetics and pharmacogenomics studies Hum Genomics 2006 2 318 328 16595076 10.1186/1479-7364-2-5-318 PMC3500181

[B5] Yoon Y Song J Hong S Kim J Analysis of multiple single nucleotide polymorphisms of candidate genes related to coronary heart disease susceptibility by using support vector machines Clin Chem Lab Med 2003 41 529 534 10.1515/CCLM.2003.080 12747598

[B6] Bureau A Dupuis J Falls K Lunetta KL Hayward B Keith TP Van Eerdewegh P Identifying SNPs predictive of phenotype using random forests Genet Epidemiol 2005 28 171 182 10.1002/gepi.20041 15593090

[B7] Díaz-Uriarte R de Andrés Alvarez S Gene selection and classification of microarray data using random forest BMC Bioinformatics 2006 7 3 10.1186/1471-2105-7-3 16398926 PMC1363357

[B8] Glaser B Nikolov I Chubb D Hamshere ML Segurado R Moskvina V Holmans P Analyses of single marker and pairwise effects of candidate loci for rheumatoid arthritis using logistic regression and random forests BMC Proc 2007 1 Suppl 1 S54 10.1186/1753-6561-1-s1-s54 18466554 PMC2367457

[B9] Lunetta KL Hayward LB Segal J Van Eerdewegh P Screening large-scale association study data: exploiting interactions using random forests BMC Genet 2004 5 32 10.1186/1471-2156-5-32 15588316 PMC545646

[B10] Meng YA Yu Y Cupples LA Farrer LA Lunetta KL Performance of random forest when SNPs are in linkage disequilibrium BMC Bioinformatics 2009 10 78 10.1186/1471-2105-10-78 19265542 PMC2666661

[B11] Nonyane B Foulkes A Application of two machine learning algorithms to genetic association studies in the presence of covariates BMC Genetics 2008 9 71 10.1186/1471-2156-9-71 19014573 PMC2620353

[B12] Sun YV Cai Z Desai K Lawrance R Leff R Jawaid A Kardia SL Yang H Classification of rheumatoid arthritis status with candidate gene and genome-wide single-nucleotide polymorphisms using random forests BMC Proc 2007 1 Suppl 1 S62 10.1186/1753-6561-1-s1-s62 18466563 PMC2367463

[B13] Breiman L Random Forests Machine Learning 2001 45 5 32 10.1023/A:1010933404324

[B14] Breiman L Friedman J Olshen R Stone C Classification and Regression Trees 1984 New York: Chapman & Hall

[B15] Breiman L Bagging Predictors Machine Learning 1996 24 123 140

[B16] Breiman L Out-Of-Bag Estimation 1996 Tech. rep., UC Berkeley

[B17] Hastie T Tibshirani R Friedman J Elements of Statistical Learning 2009 2 New York: Springer

[B18] Genuer R Poggi JM Tuleau C Random Forests: some methodological insights Tech rep, INRIA 2008 http://hal.inria.fr/inria-00340725/en/

[B19] Hafler DA Compston A Sawcer S Lander ES Daly MJ De Jager PL de Bakker PI Gabriel SB Mirel DB Ivinson AJ Pericak-Vance MA Gregory SG Rioux JD McCauley JL Haines JL Barcellos LF Cree B Oksenberg JR Hauser SL Risk alleles for multiple sclerosis identified by a genomewide study N Engl J Med 2007 357 851 862 10.1056/NEJMoa073493 17660530

[B20] Browning SR Browning BL Rapid and accurate haplotype phasing and missing-data inference for whole-genome association studies by use of localized haplotype clustering Am J Hum Genet 2007 81 1084 1097 10.1086/521987 17924348 PMC2265661

[B21] Purcell S Neale B Todd-Brown K Thomas L Ferreira MA Bender D Maller J Sklar P de Bakker PI Daly MJ Sham PC PLINK: a tool set for whole-genome association and population-based linkage analyses Am J Hum Genet 2007 81 559 575 10.1086/519795 17701901 PMC1950838

[B22] Liaw A Wiener M Classification and regression by randomForest Rnews 2002 2 18 22

[B23] Salford Systems http://salford-systems.com/

[B24] Svetnik V Liaw A Tong C Intrator N, Masulli F Variable selection in random forest with application to quantitative structureactivity relationship Proceedings of the 7th Course on Ensemble Methods for Learning Machines Springer-Verlag

[B25] Oksenberg JR Barcellos LF Multiple sclerosis genetics: leaving no stone unturned Genes Immun 2005 6 375 387 10.1038/sj.gene.6364237 15973459

[B26] Strobl C Boulesteix AL Zeileis A Hothorn T Bias in random forest variable importance measures: illustrations, sources and a solution BMC Bioinformatics 2007 8 25 10.1186/1471-2105-8-25 17254353 PMC1796903

[B27] Pearson TA Manolio TA How to interpret a genome-wide association study JAMA 2008 299 1335 1344 10.1001/jama.299.11.1335 18349094

[B28] Australia, Consortium NZMSG Genome-wide association study identifies new multiple sclerosis susceptibility loci on chromosomes 12 and 20 Nature Genetics 2009 41 824 828 10.1038/ng.396 19525955

[B29] Ward GR Franklin SO Gerald TM Dempsey KT Clodfelter DE Krissinger DJ Patel KM Vrana KE Howlett AC Glucocorticoids plus opioids up-regulate genes that influence neuronal function Cell Mol Neurobiol 2007 27 651 660 10.1007/s10571-007-9151-3 17554624 PMC11517204

[B30] deJager PL Jia X Wang J deBakker PIQ Ottoboni L Aggarwal NT Piccio L Raychaudhuri S Tran D Aubin C Briskin R Romano S IMSGC Meta-analysis of genome scans and replication identify CD6, IRF8 and TNFRSF1A as new multiple sclerosis susceptibility loci Nature Genetics 2009 41 776 782 10.1038/ng.401 19525953 PMC2757648

[B31] Morgan AR Hamilton G Turic D Jehu L Harold D Abraham R Hollingworth P Moskvina V Brayne C Rubinsztein DC Lynch A Lawlor B Gill M O'Donovan M Powell J Lovestone S Williams J Owen MJ Association analysis of 528 intra-genic SNPs in a region of chromosome 10 linked to late onset Alzheimer's disease Am J Med Genet B Neuropsychiatr Genet 2008 147B 727 731 10.1002/ajmg.b.30670 18163421

[B32] Wider C Lincoln SJ Heckman MG Diehl NN Stone JT Haugarvoll K Aasly JO Gibson JM Lynch T Rajput A Rajput ML Uitti RJ Wszolek ZK Farrer MJ Ross OA Phactr2 and Parkinson's disease Neurosci Lett 2009 453 9 11 10.1016/j.neulet.2009.02.009 19429005 PMC2684848

[B33] van Roon JA Lafeber FP Role of interleukin-7 in degenerative and inflammatory joint diseases Arthritis Res Ther 2008 10 107 10.1186/ar2395 18466642 PMC2453758

